# Pre-Asylum Treatment

**Published:** 1910-07-02

**Authors:** 


### PRE-ASYLUM TREATMENT.

At the annual meeting of the directors of the Dundee Royal Asylum Dr. J. Mackie Whyte announced that of twenty-eight cases treated in this way eighteen were improved and ten were not. In several cases the benefit was great. There had been two or three recoveries from a distinctly insane state. These cases were treated on probation, as it were, and appear not to have been formally admitted into the asylum. Such a method of treatment is eminently desirable, and it would be a good thing were it widely adopted, but the Scotch law is very different from ours, and persons who are distinctly insane may not be detained under care and treatment in this country except under certificate and a magistrate's order.

If the recommendations of the Royal Commission were adopted, detention would become easier, and require, in many cases, fewer formalities; and temporary detention for six months would not necessitate the machinery now required. That many persons who are mentally affected would benefit by earlier treatment, and that early treatment is often postponed because of the horror of asylums, until it becomes late treatment, and the improvable stage is passed, is unfortunately true, especially among the wealthier classes. The poor have to a great extent lost their horror of asylums. They know too much of the comfort and care of asylum life to retain the old dread. The immense number of lunatics now in asylums insures that a very large proportion of the population has visited some relative or friend in an asylum, or knows someone who has done so, and the objections to asylums are becoming dissipated.

Among the well-to-do much of the old prejudice still remains, and some of the neglect of early and appropriate treatment is due to this cause; but much of it is due to the fact that the neurologists, who are very frequently consulted in mental cases, almost invariably send these patients into nursing homes, where the conditions are not adapted to the treatment of mental disorder, and advise a rest-cure, which is often extremely inappropriate treatment. People have a horror not only of asylums but of consulting "mental specialists," and often potter about with inappropriate treatment until the chances of recovery are, to say the least, seriously diminished. How an operating surgeon can reconcile it with his conscience to undertake the treatment of cases of mental disease is difficult to understand, for the undertaking is at least as much *ultra vires*, and as likely to be disastrous, as it would be for the "mental specialist" to perform an operation for appendicitis or for cataract. A celebrated surgeon of the last generation, when asked to define a surgical case, gave as his definition, "Any case that comes into my consulting-room," and the definition has had a prolonged vitality.

